# Treatment of Typhoid Fever

**Published:** 1892-09

**Authors:** 


TREATMENT OF TYPHOID FEVER.—In a paper read before the Florida Medical Association (*Transactions*, 1892,) Dr. Warren E. Anderson, of Pensacola, states his belief that typhoid fever being a self-limiting disease, attempts to abort it are injurious. He cautiously administers, with most gratifying results, minute doses of calomel throughout the entire course of cases. Its antiseptic properties prevent rapid decomposition of the ingesta; while its stimulating effect upon the kidneys and glandular system hastens the elimination of the poison from the system. He thinks oil of turpentine superior to salol, hydro-naphthol, etc., as an intestinal antiseptic—the most delicate stomach soon acquiring a tolerance of it. Quinine is of unquestionable value, but do not keep the patient cinchonized. *Do not resort to antipyretics* until the temperature rises above 102 degs. F. Where an antipyretic is required, he prefers antikamnia Cold water sponging of the neck, face and upper extremities is grateful to many patients. In the ten recent cases in which he has used hot-water enemata, flushing the colon, he has been more than pleased with the results. He has been repaid by allowing copious draughts of water—not warm until convalescence, nutrition should consist of boiled milk, with freshly expressed juice of beef.

Dr. Joseph D. Rush, of Apalachicola, Fla., (*Idem*) reports favorably on the value of salol in typhoid fever. He details two cases. One, a married lady, brunette, age 24 years, with fever reaching 105 degs. F. by the seventh day, after the failure of calomel, soda and quinia, was given the following:

R. Salol.
Antikamnia....................................aa dr. ss.
Quiniæ sulph.................................gr. vj.
Mix. Make twelve capsules. Sig. One every three hours.

This prescription was kept up twelve days, when convalescence began. Alcoholic baths to the spinal column were given once a day. Diet was strictly boiled milk and beef tea.

The other case was that of a colored boy, age 13 years, temperature 105 deg. Same prescription, sponging and diet as above. Recovered. His conclusions are that salol as an internal antiseptic, with daily sponging of the body with alcohol, and with a diet of hot boiled milk, promises much in the treatment of low and continued fevers, with bowel complications.—*Virginia Medical Monthly.*

